# Polymorphism in the oxytocin promoter region in patients with lactase non-persistence is not related to symptoms

**DOI:** 10.1186/1471-230X-9-90

**Published:** 2009-11-30

**Authors:** Mikael Truedsson, Joyce Carlson, Magnus Simrén, Bodil Ohlsson

**Affiliations:** 1 Department of Clinical Sciences, Division of Gastroenterology and Hepatology, Malmö University Hospital, Lund University, Lund, Sweden; 2 Labmedicine Skåne, Clinical Chemistry Lund, Lund University, Lund, Sweden; 3 Department of Internal Medicine, Division of Gastroenterology and Hepatology, Sahlgrenska University Hospital, Gothenburg University, Gothenburg, Sweden

## Abstract

**Background:**

Oxytocin and the oxytocin receptor have been demonstrated in the gastrointestinal (GI) tract and have been shown to exert physiological effects on gut motility. The role for oxytocin in the pathophysiology of GI complaints is unknown. The aim of this study was to examine genetic variations or polymorphism of oxytocin (*OXT*) and its receptor (*OXTR*) genes in patients with GI complaints without visible organic abnormalities.

**Methods:**

Genetic variants in the *OXT *promoter region, and in the *OXTR *gene in DNA samples from 131 rigorously evaluated patients with Irritable Bowel Syndrome (IBS), 408 homozygous subjects referred for lactase (LCT-13910 C>T, rs4988235) genotyping, and 299 asymptomatic blood donors were compared. One polymorphism related to the *OXT *gene (rs6133010 A>G) and 4 related to the *OXTR *gene (rs1465386 G>T, rs3806675 G>A, rs968389 A>G, rs1042778 G>T) were selected for genotyping using Applied Biosystems 7900 HT allele discrimination assays.

**Results:**

There were no statistically significant differences in the genotype or allele frequencies in any of the SNPs when IBS patients were compared to healthy controls. Among subjects referred for lactase genotyping, the rs6133010 A>G *OXT *promoter A/G genotype tended to be more common in the 154 non-persistent (27.3%) subjects than in the 254 lactase persistant (18.1%) subjects and in the healthy controls (19.4%) (p = 0.08). When direct comparing, the A/G genotype was less common in the *OXT *promoter region in controls (p = 0.09) and in subjects with lactase persistence (p = 0.03) compared to subjects with lactase non-persistence. When healthy controls were viewed according to their own LCT-13910 genotypes, the C/C lactase non-persistent controls had a higher frequency for the *OXT *promoter A/G genotype than LCT-13910 T/T lactase persistent controls (41.2% vs 13.1%).

No significant differences in frequencies of the investigated *OXTR *SNPs were noted in this study.

**Conclusion:**

The results suggest that polymorphism in the promoter region of the *OXT *gene is most common in subjects with lactase non-persistence. This polymorphism may not be related to GI symptoms, as it is related to lactase non-persistence also in healthy controls.

## Background

Gastrointestinal (GI) complaints in the form of abdominal pain and altered bowel movements are very common in the population, and in most cases, remain unexplained despite extensive diagnostic evaluations. These patients are classified as suffering from functional bowel diseases [1]. Frequently indistinguishable symptoms are seen in the subgroup of patients with lactase non-persistence, which is also common in the adult population [2,3], and reported complaints in this group are surprisingly less correlated to the volume of milk intake, than previously assumed [4]. Differences in gastric emptying and individual perception of symptoms have been discussed [5,6]. Many neuropeptides, e.g. serotonin and norepinephrine, are involved in the regulation of GI sensitivity and motility, and polymorphism in these peptides has been suggested as another possible explanation to symptoms [7-9].

Oxytocin was long thought to be a hormone primarily involved in parturition and suckling. Recently, oxytocin expression has been found in the gut [10,11], where it is secreted after a fatty meal [12] and stimulates colonic activity [13]. It has further been demonstrated that systemic administration of oxytocin leads to enhanced gastric emptying [14,15] and that the oxytocin receptor antagonist atosiban significantly delays gastric emptying [16]. In accordance to this, diabetic patients with gastro paresis have no demonstrable postprandial oxytocin release [17].

The aim of this study was thus to examine whether genetic variation or polymorphism in the *OXT *or *OXTR *genes may explain the pathology in numerous patients with GI complaints without organic explanation.

## Methods

This study was performed according to the Helsinki declaration and approved by the Ethics Committee of Lund University.

### Study Population

One hundred and thirty-one patients (108 females), mean age 38.2 ± 15.1 years, range 20-78 years, who have undergone rigorous evaluations for GI complaints at tertiary care centres in Malmö (N = 43) and in Gothenburg (N = 88) were included in the study. LCT genotyping was performed, and 13 patients were found to have LCT-13910 C/C, 76 patients had LCT-13910 T/T and 38 patients were heterocygotes. In the absence of evident organic disease, and as the patients with LCT-13910 C/C had persistent GI complaints after elimination of milk products in the food, they were regarded as having irritable bowel syndrome (IBS) (1, 3). They had predominantly constipation (N = 35), diarrhoea (N = 35) or mixed symptoms (N = 59), whereas clinical subgroups were not defined for 2 patients. Samples and written informed consent for this study were obtained from these patients and from 299 healthy adult blood donors (99 females), mean age 64.5 ± 13.0 years, range 41-85 years, who negated all forms of abdominal pain or abnormal bowel habits on a written questionnaire.

Remnant samples submitted from an additional 319 subjects from predominantly primary care centres to screen for potential lactase non-persistence by analysis of LCT-13910C>T (rs4988235), and who had had homozygous C/C or T/T genotypes were also included in this pilot study. After initial registration of age, sex and genotype, all personal identifiers associated with the samples were destroyed. This procedure is in accordance with the Swedish biobank law. These samples were put together with the 89 patients from tertiary care centres with either homocygous for C/C or T/T. Of these totally 408 subjects, 154 (mean age 30.5 ± 18.2 years, range 3-76 years, 113 females) were LCT-13910 C/C homozygotes for lactase non-persistence and 254 (mean age 36.8 ± 19.3 years, range 2-81 years, 189 females) were LCT-13910 T/T homozygotes for lactase persistence. Heterozygous C/T samples were excluded from this study to avoid ambiguity in interpretation. Although the samples were not consistently tested for adjacent LCT variants, e.g. LCT-13907 C>G and LCT-13915 T>G, these when rarely present, are frequently noted outside of the main LCT-13910 C/C or C/T clusters. This is seen in 1-2% of samples at our laboratory and was not expected to interfere with interpretations of our results.

### SNP selection

The *OXT *gene is located on chromosome 20p13 [18] and the *OXTR *gene is located on chromosome 3p26 [19]. Some transcript variants have been identified in these genes. We conducted an extensive search using Entrez SNP [20] and HapMap genome browser [21] databases for known SNPs in and immediately up- and downstream of the *OXT *and *OXTR *genes. Five SNPs with heterozygosity of ≥ 5% were identified; the *OXT *promoter region (rs61333010 A>G), the *OXTR *5'promoter region (rs3806675 G>A), the *OXTR *5'hap marker (rs1465386 G>T), the *OXTR *exon 1 (rs1042778 G>T) and the *OXTR *exon 4 (rs968389A>G).

### DNA analysis

DNA was extracted from fresh EDTA whole blood using QiaAmp minikits (Qiagen GmbH, Germany) according to the manufacturer's instructions. Genotyping of all SNPs was performed according to the standard protocol at the Department of Clinical Chemistry at Malmö University Hospital, using commercially available SNP genotyping (Applied Biosystems) on an Applied Biosystems 7900 HT instrument, no UNG mastermix and the recommended PCR program. Specific assays were C_30100478_10 for rs6133010, C_7622000_0 for rs968389, and C_7622140_30 for rs1042778. Assays by design were acquired for rs4988235, rs14653886 and rs3806675. All samples, including the primary care subjects were then analysed with all assays in the LCT, *OXT *and *OXTR *genes.

### Statistical analyses

Fisher's exact test was used for comparison between groups, and odds ratio (OR) and confidence interval (CI) were calculated. Mann Whitney U test was used for calculations of differences in ages between groups. P < 0.05 was considered statistically significant.

## Results

When comparing IBS patients from tertiary care centres to healthy controls, no overall statistical significant differences were observed in genotype or allele frequencies (data not shown). There was no association between symptoms and genotype in any of the selected SNPs in the *OXT *and *OXTR *genes (Table 1 and 2, other data not shown).

**Table 1:** Genotype frequency of the oxytocin (*OXT*) promoter region rs6133010 A>G in patients, subjects and controls

Genotype	A N (%)	A/G n (%)	G/G N (%)	P value
Constipation-predominated IBS (n:35)	30 (85.7)	5 (14.3)	0 (0.0)	

Mixed IBS (n:59)	49 (83.1)	10 (16.9)	0 (0.0)	P = 0.60*

Diarrhoea-predominated IBS (n:35)	26 (74.3)	9 (25.7)	0 (0.0)	

				

Lactase persistence (n:254)	208 (81.9)	46 (18.1)	0 (0.0)	

Lactase non-persistence (n:154)	111 (72.1)	42 (27.3)	1 (0.6)	P = 0.08**

				

Controls (n:299)	240 (80.3)	58 (19.4)	1 (0.3)	

* = Comparisons made between subgroups of patients with Irritable Bowel Syndrome (IBS).

** = Comparisons made between controls and lactase persistent- and lactase non-persistent subjects.

Fisher's exact test.

**Table 2:** Genotype frequency of the oxytocin receptor (*OXTR*) 5'promoter region rs3806675 G>A in patients with lactase non-persistence or lactase persistence

Genotype	G/G n (%)	A/G n (%)	A/A n (%)	P value
Constipated-dominated IBS (n:34)	11 (32.4)	18 (52.9)	5 (14.7)	

Mixed IBS (n:59)	15 (25.4)	33 (55.9)	11 (18.6)	P = 0.44*

Diarrhoea-dominated IBS (n:35)	13 (37.1)	20 (57.1)	2 (5.7)	

				

Lactase persistence (n:254)	69 (27.2)	128 (50.4)	56 (22.0)	

Lactase non-persistence (n:154)	55 (35.7)	76 (49.4)	22 (14.3)	P = 0.07**

				

Controls (n:299)	101 (33.8)	146 (48.8)	52 (17.4)	

* = Comparisons made between subgroups of patients with Irritable Bowel Syndrome (IBS).

** = Comparisons made between controls and lactase persistent- and lactase non-persistent subjects.

Fisher's exact test.

As genotypes in patients with known IBS did not differ from controls, we examined whether there were any differences between subjects with or without lactase persistence, according to their *OXT *or *OXTR *genotypes. There was no difference in age between the lactase non-persistent or lactase persistent group (p = 0.39), whereas there was a significantly higher age in the controls compared to both subject groups (p < 0.00). When the whole study population was included, there was no difference between females and males, or between presumably pre- or postmenopausal subjects (age < or > 50 years of age) in relation to the selected SNPs in the *OXT *and *OXTR *genes (data not shown).

When the controls were compared to lactase-persitent and lactase non-persistent subjects, there was a tendency to significance in the genotype frequency in the OXT promoter region (rs6133010 A>G) (Table 1). When comparing allele frequencies, there was no significant difference (Table 3). Calculation of odds ratio and confidence interval for the allele frequencies showed no difference between controls and lactase persistent subjects (OR: 0.89; CI 0.50-1.58; P = 0.77), or between controls and lactase non-persistent subjects (OR: 1.49; CI: 0.83-2.69; P = 0.21).

**Table 3:** Allele frequencies of the oxytocin (OXT) promoter region rs6133010 A>G in controls and in subjects with lactase non-persistence or lactase persistence

	A n (%)	G n (%)
Lactase persistence (n:254)	231 (90.9)	23 (9.1)

Lactase non-persistence (n:154)	132 (85.7)	22 (14.3)

		

Controls (n:299)	269 (90.0)	30 (10.0)

Fisher's exact test. P = 0.24

Although there was no difference in allele frequencies for rs6133010 A>G (OR: 0.60; CI: 0.32-1.11; P = 0.11) between LCT-13910 C/C or T/T subjects, T/T (lactase persistent) subjects were less likely to have the A/G genotype for this SNP in the *OXT *promoter region (P = 0.03). A slightly lower frequency of this A/G genotype was also seen in healthy controls compared to lactase non-persistent (LCT-13910 C/C) subjects (P = 0.09), with no difference between controls and lactase persistent subjects (P = 0.86). The frequency of the A/A genotype in the different study groups is shown in Table 1. When regarding the asymptomatic blood donors according to their LCT genotype, the frequency of LCT-13910 C/C was 8%, similar to the IBS patients from the tertiary care centre (10%). The lactase non-persistent asymptomatic controls had a frequency of the rs6133010 A/G genotype in 41%, compared to 13% in the lactase persistent controls.

In the OXTR 5'promoter region (rs3806675 G>A) there was a trend towards higher A/A genotype in relation to G/G genotype in lactase persistent subjects compared to non-persistent subjects (Table 2), although allele frequencies did not differ between the groups (OR: 0.72; CI: 0.48-1.08; P = 0.12).

There were no differences in genotype or allele frequencies in the other parts of OXT or OXTR genes examined (data not shown).

## Discussion

Among patients with unexplained GI complaints referred for lactase (LCT-13910 C>T) genotyping, a significant difference was found in genotype frequency of a SNP in the *OXT *promoter region gene between LCT-13910 C/C (non-persistent) and T/T (persistent) subjects. This polymorphism was observed also in asymptomatic controls when these were divided according to C/C and T/T.

The symptoms leading to admission for lactase genotyping is abdominal complaints after lactose intake in the form of abdominal pain and bloating, diarrhoea and an excess of gas. These symptoms are often impossible to differ from functional bowel diseases without making a genotyping. Patients referred for lactase genotyping who do not carry the lactase non-persistence genotype, may in the majority of cases suffer from functional bowel diseases [1]. Even among lactase non-persistence subjects, many patients suffer from functional bowel diseases, and although reducing the milk intake, the symptoms persist [4]. In the present study, we chose only homozygote patients to obtain well-defined groups concerning lactase persistence, as individual variations in the lactose absorption, particularly among heterozygotes have been reported [2,4]. Factors causing this variation of symptoms in response to the same volume of milk intake, or according to different milk products, may include the amount of lactose not absorbed in the gut, differences in the bacterial flora and their metabolism of lactose in colon [22], and the rate of gastric emptying and individual perception of symptoms [5,6]. In the present study, the A/A genotype of rs6133010 in the promoter region of the *OXT *gene was more frequent in asymptomatic controls and in lactase persistent subjects than in lactase non-persistent, although statistically significant only in persistent subjects. This suggests that the A/A genotype regulates the GI physiology in a different way compared to A/G. However, when comparing the LCT-13910 genotype in asymptomatic controls, C/C homozygous controls had the highest A/G frequency of all groups. Although the small sample size, this finding suggests that A/G may not be an important factor for the symptomatology, but is coupled to other factors such as LCT-13910 C/C.

During the last years, further genotypes associated with lactase persistence/non-persistence, e.g. LCT-13907C>G and LCT-13915T>G, have been described [23]. These genotypes were not tested for in the actual study. However, as these genotypes are uncommon, and only a minority of the population in Sweden comes from the part of the world where these genotypes are most frequent, this may not obscure the picture.

Previously it has been suggested that oxytocin play a role in the regulation of GI physiology as it has been found in both the myenteric and submucous ganglia in the human GI tract [11]. It is released in response to a fatty meal [12] and has been shown to accelerate gastric emptying [14,15], whereas the oxytocin receptor antagonist atosiban inhibited gastric emptying [16]. Furthermore, oxytocin stimulated colonic peristalsis in healthy women [13]. On the other hand, studies in animals have shown that oxytocin inhibited gastric emptying [24] and contractile motility of the proximal [25] and distal colon [26]. Possible explanation to these divergent results are differences in methods i.e.; dose and administration of oxytocin, in species studied, or in the region of the GI tract examined. Nothing has been described about the role of oxytocin in the pathophysiology of disturbances of GI motility.

Abdominal pain is the most frequent complaint among GI disturbances. Continuous infusion of oxytocin significantly elevated thresholds for visceral perception of colonic discomfort in patients with IBS [27], and increased the threshold for peripheral pain in rats [28]. Nasal administration of oxytocin had a positive effect on abdominal pain in patients with IBS [29]. Altogether, this suggests oxytocin to be involved in pain regulation, which is further supported by the expression of the peptide in the dorsal root ganglia [30] and its widely spread presence in the central nervous system [31].

Estradiol regulates the *OXT*-gene expression in rat uterus [32]. Recently, it has been shown that estradiol upregulates the oxytocin receptor in the enteric plexus of colon in rats and increases the colon sensitivity to the excitatory effect of oxytocin on colon motility [33]. Females have higher levels of oxytocin and estradiol than men [34], and IBS is more common in females than in men [35]. In female patients suffering from IBS, blood estradiol and oxytocin levels were significantly lower than in control groups [36,37]. As many GI complaints debute at an age when the estradiol levels fall, lower expression of oxytocin receptors has been one hypothesis for the etiology of GI disturbances. However, differences in hormonal plasma levels may be secondary, and not causal. When comparing the genotype frequency in IBS patients in the table over rs6133010 A>G, the *OXT *gene promoter region which is closely coupled to estradiol [32,38], it seems as though patients with mixed IBS have similar frequencies of the genotypes as healthy controls, and diarrhoea-dominated IBS patients resemble subjects with lactase non-persistence. This finding raises the hypothesis that the symptoms in patients with lactase non-persistence, irrespective of milk intake, may depend on genetic factors resembling IBS patients with diarrhoea. This should be further evaluated in larger studies. However, we could not find any association between gender and polymorphism in the examined SNPs.

A limitation of this pilot study is the small sample size, and our lack of information concerning specific symptoms in subjects referred for LCT-13910 genotyping from the primary care centres. As people from northern Europe have a higher prevalence of lactase persistence than people from southern Europe, Africa and Asia [39], we can assume that the group of lactase non-persistent subjects contained a larger proportion of subjects with a non-Nordic background. These differences in ethnicity can possibly explain the genetic variation to some extent. Alternatively, the *OXT *promoter G allele may lead to increased pain, and thus increased medical evaluation, in patients with lactase non-persistence.

## Conclusion

Our investigation has shown significant differences in genotype frequencies of a SNP in the promoter region of the *OXT *gene in patients with lactase non-persistence. The role of this polymorphism in the pathophysiology and symptomatology of GI complaints remains obscure, as the polymorphism was also present in healthy LCT C/C homozygous controls.

## Abbreviations

GI: gastrointestinal; OXT: oxytocin; OXTR: oxytocin receptor.

## Competing interests

The authors declare that they have no competing interests.

## Authors' contributions

MT has substantial contributions to conception and design, analysis and interpretion of data and mainly drafting the manuscript. JC has contributed to conception and design, acquisition of data, analysis and interpretion of data and has been involved in revising the manuscript critically. MS has contributed to analysis and interpretion of data and have been involved in revising the manuscript critically. BO has contributed to conception and design, acquisition of data, analysis and interpretion of data and has been involved in drafting the manuscript. All authors have read and approved the final manuscript.

## Authors' information

MT is PhD student and Specialist in Internal Medicine, JC is Associated professor and Senior Consultant of Gastroenterology and Hepatology, MS is Associated professor and Senior Consultant of Gastroenterology and Hepatology, BO is Professor in Medicine and Senior Consultant of Gastroenterology and Hepatology

## Pre-publication history

The pre-publication history for this paper can be accessed here:

http://www.biomedcentral.com/1471-230X/9/90/prepub
